# Carbogen-induced changes in rat mammary tumour oxygenation reported by near infrared spectroscopy

**DOI:** 10.1038/sj.bjc.6690272

**Published:** 1999-04

**Authors:** E L Hull, D L Conover, T H Foster

**Affiliations:** Departments of Radiology and ofBiochemistry and Biophysics, University of Rochester School of Medicine and Dentistry, 601 Elmwood Ave., Rochester, 14642, NY, USA; Department of Physics and Astronomy, University of Rochester, Rochester, NY, 14627, USA

**Keywords:** oxygen, tumour oxygenation, carbogen, haemoglobin, diffuse reflectance spectroscopy, radiotherapy

## Abstract

We have evaluated the ability of steady-state, radially-resolved, broad-band near infrared diffuse reflectance spectroscopy to measure carbogen-induced changes in haemoglobin oxygen saturation (SO_2_) and total haemoglobin concentration in a rat R3230 mammary adenocarcinoma model in vivo. Detectable shifts toward higher saturations were evident in all tumours (*n* = 16) immediately after the onset of carbogen breathing. The SO_2_ reached a new equilibrium within 1 min and remained approximately constant during 200–300 s of administration. The return to baseline saturation was more gradual when carbogen delivery was stopped. The degree to which carbogen increased SO_2_ was variable among tumours, with a tendency for tumours with lower initial SO_2_ to exhibit larger changes. Tumour haemoglobin concentrations at the time of peak enhancement were also variable. In the majority of cases, haemoglobin concentration decreased in response to carbogen, indicating that increased tumour blood volume was not responsible for the observed elevation in SO_2_. We observed no apparent relationship between the extent of the change in tumour haemoglobin concentration and the magnitude of the change in the saturation. Near infrared diffuse reflectance spectroscopy provides a rapid, non-invasive means of monitoring spatially averaged changes in tumour haemoglobin oxygen saturation induced by oxygen modifiers. © 1999 Cancer Research Campaign


*British Journal of Cancer* (1999) **79**(11/12), 1709–1716
© 1999 Cancer Research Campaign
Article no. bjoc.1998.0272

### Carbogen-induced changes in rat mammary tumour oxygenation reported by near infrared spectroscopy

**EL Hull[3], DL Conover[1] and TH Foster[1,2,3]**

Departments of [1]Radiology and of [2]Biochemistry and Biophysics, University of Rochester School of Medicine and Dentistry, 601 Elmwood Ave., Rochester, NY 14642, USA; [3]Department of Physics and Astronomy, University of Rochester, Rochester, NY 14627, USA

**Summary** We have evaluated the ability of steady-state, radially-resolved, broad-band near infrared diffuse reflectance spectroscopy to measure carbogen-induced changes in haemoglobin oxygen saturation ($SO_2$) and total haemoglobin concentration in a rat R3230 mammary adenocarcinoma model in vivo. Detectable shifts toward higher saturations were evident in all tumours ($n$ = 16) immediately after the onset of carbogen breathing. The $SO_2$ reached a new equilibrium within 1 min and remained approximately constant during 200–300 s of administration. The return to baseline saturation was more gradual when carbogen delivery was stopped. The degree to which carbogen increased $SO_2$ was variable among tumours, with a tendency for tumours with lower initial $SO_2$ to exhibit larger changes. Tumour haemoglobin concentrations at the time of peak enhancement were also variable. In the majority of cases, haemoglobin concentration decreased in response to carbogen, indicating that increased tumour blood volume was not responsible for the observed elevation in $SO_2$. We observed no apparent relationship between the extent of the change in tumour haemoglobin concentration and the magnitude of the change in the saturation. Near infrared diffuse reflectance spectroscopy provides a rapid, non-invasive means of monitoring spatially averaged changes in tumour haemoglobin oxygen saturation induced by oxygen modifiers.

**Keywords**: oxygen; tumour oxygenation; carbogen; haemoglobin; diffuse reflectance spectroscopy; radiotherapy

The use of commercial, needle-based, oxygen-sensitive electrode systems in human trials during the past decade has provided a wealth of new information regarding the role of tumour oxygen status in predicting survivability and local recurrence. Vaupel et al (1991), for example, have shown that, in the breast, severe hypoxia is present only in malignant disease. Further, the presence of hypoxia is not related to the stage or pathologic grade of the tumour, suggesting that even if these were determined from biospy samples, no prediction regarding the presence or absence of radiation-therapy-limiting hypoxia could be made. Several studies in various anatomic sites have demonstrated a correlation between tumour hypoxia and response to treatment with ionizing radiation therapy (Höckel et al, 1993*a*; Okunieff et al, 1993; Brizel et al, 1997). In these studies, extreme hypoxia, as determined by analysis of $pO_2$ histographs obtained from measurements using polarographic needle electrodes, indicated poor prognosis. In addition to predicting local treatment failure, a recent report by Brizel et al (1996) suggested that hypoxia in primary soft tissue sarcomas was correlated with the probability of appearance of remote metastatic disease. Studies in cell culture support the suggestion that exposure to sustained hypoxia results in the selection of malignant cells expressing aggressive phenotypes (Graeber et al, 1996).

The sustained interest in tumour hypoxia has given rise to the introduction of various methods of improving tumour oxygenation. The use of hyperbaric oxygen was an early example (Thomlinson, 1960), and it continues to be studied in laboratory animal systems and in human clinical trials (Brizel et al, 1995; Voûte et al, 1995). Inhalation of high oxygen content gases such as carbogen has also been studied rather extensively (Olive and Inch, 1973; Hill and Bush, 1977; Grau et al, 1992). Chemical agents such as nicotinamide, which is designed to modify tumour blood flow, have been used either alone (Horsman et al, 1989) or together with carbogen inhalation (Rojas, 1992) in an effort to sensitize tumour cells rendered hypoxic by more than one mechanism (i.e. chronic vs acute). The combination of nicotinamide and carbogen in conjunction with accelerated radiotherapy is being evaluated currently in clinical trials in Europe (Saunders and Dische, 1996). Each of these methods has demonstrated some success in specific situations and tumour models, while none has emerged yet as being generally accepted in clinical practice.

The importance of hypoxia in predicting tumour response to ionizing radiation (and photodynamic) therapy, and in possibly predicting the likelihood of metastases, has stimulated great interest in improving techniques for clinical measurement of tumour oxygenation in vivo. These methods and others suitable only for laboratory use have been reviewed recently by Stone et al (1993). While polarographic electrode systems have come to be viewed as a standard, their widespread, routine clinical acceptance may be limited by their invasiveness (Saunders and Dische, 1996). This may be especially important if tumour oxygenation is to be monitored repeatedly during the course of fractionated therapy.

Near infrared spectroscopy is among the non-invasive methods that are sensitive to some measure of tumour oxygenation. Specifically, because the absorption spectra of deoxy- and oxyhaemoglobin are markedly different, the haemoglobin oxygen

*Received 21 April 1998*
*Revised 19 August 1998*
*Accepted 21 August 1998*

*Correspondence to:* TH Foster

($HbO_2$) saturation may be determined from the tissue absorption spectrum, which may be reconstructed from appropriate reflectance measurements. Successful spectroscopic determination of the $HbO_2$ saturation ($SO_2$) in the near infrared spectral region depends upon two things: (1) the ability to determine accurately the absorption spectrum in the presence of significant light scattering by tissue and (2) the ability to account properly for the contributions of chromophores other than haemoglobin in the measured tissue absorption spectrum. We have recently reported on a multi-wavelength, continuous-wave diffuse reflectance spectrometer and data reduction scheme that address both of these criteria (Hull and Foster, 1997; Nichols et al, 1997; Hull et al, 1998). Here, we describe the use of this spectroscopic method to obtain non-invasively the response of subcutaneous rat mammary adenocarcinomas to carbogen breathing. The technique is simultaneously sensitive to both the $HbO_2$ saturation and to the tumour haemoglobin concentration.

### MATERIALS AND METHODS

### Near infrared spectroscopy

Our instrumentation and method of data reduction for performing quantitative diffuse reflectance spectroscopy in highly scattering systems has been described in detail elsewhere (Nichols et al, 1997; Hull et al, 1998). Briefly, we have constructed a continuous wave spectrometer similar in design to that first suggested by Wilson et al (1990). Broad-band light from a mercury arc lamp is delivered to the surface of the tissue via a 400 μm core diameter optical fibre (the source fibre), which is terminated in a probe of our own design and construction. The probe also holds a linear array of 20 detection fibres (200 μm core diameter) located at distances of 1.0–20.0 mm from the source fibre (see Figure 1). During a measurement, the probe is placed in direct contact with the skin overlying the tumour. Light from the source fibre is injected into the tissue, and the diffusely scattered reflectance is collected by the 20 detection fibres at known distances from the injection point. Monte Carlo simulations demonstrate that the average depths of the light paths from the source optical fibre to the most remote detection fibres (which determine the absorption coefficient) are approximately 3–4 mm. The output of these detection fibres is then imaged through a grating spectrograph (SpectraPro®-275i, Acton Res. Corp, Acton, MA, USA) onto the surface of a liquid nitrogen-cooled CCD camera (Princeton Inst., Princeton, NJ, USA). The imaging spectrograph preserves the positions of the detection fibres at the detector, and the dispersion of the grating and active area of the detector are such that 164 nm of spectral information is recorded simultaneously. Typical signal integration times for measurements described here are approximately 5 s.

The spatially and spectrally resolved signal at the detector is treated as a set of separate, radially resolved diffuse reflectance curves at each of the 164 wavelengths. Each diffuse reflectance data set is analysed using an expression derived from the diffusion theory approximation to radiative transport described by Haskell et al (1994) and by Kienle and Patterson (1997). Fitting this expression to the spatially resolved diffuse reflectance allows the absorption coefficient and the transport scattering coefficient of the medium at one wavelength to be determined independently. Repeating this analysis for each wavelength over the full detected spectral range enables the reconstruction of an absorption spectrum that is uncorrupted by the effects of multiple light scattering.

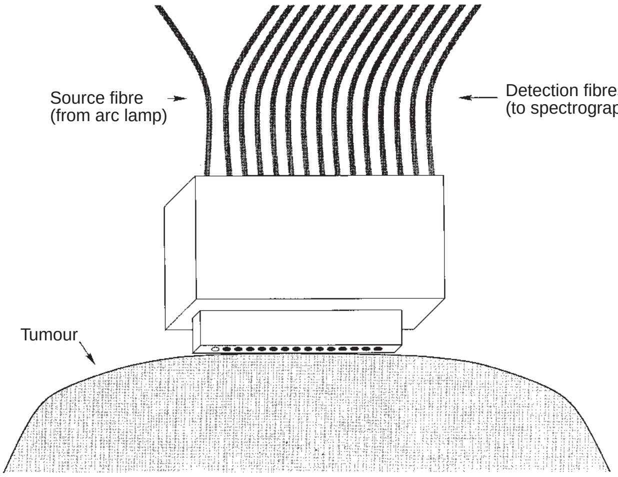

**Figure 1** A schematic drawing of the optical probe in contact with the tumour surface. A total of 20 detection fibres are located at distances of 1.0–20.0 mm from the source fibre

Once the absorption spectrum has been constructed in this way, the oxy- and deoxyhaemoglobin concentrations in the tissue are determined using a singular value decomposition (SVD) algorithm adapted from Press et al (1992). This approach has been described elsewhere (Hull and Foster, 1997; Hull et al, 1998). Briefly, the net absorption coefficient from the tissue is treated as a sum of contributions from oxyhaemoglobin, deoxyhaemoglobin, and from the remaining non-haemoglobin absorbers. In its present implementation, these other absorbers were not specified explicitly, and their contribution to the tissue absorption was represented by an expansion in terms of sines and cosines where the amplitudes are returned by the SVD routine. In practice, a total of 10 sine and 10 cosine terms is sufficient to reconstruct the background, non-haemoglobin absorption. The tissue absorption spectrum, $\mu_a(\lambda)$, is expressed,

$$\mu_a(\lambda) = \epsilon_{Hb}(\lambda)[Hb] + \epsilon_{HbO_2}(\lambda)[HbO_2] + A_0 + \sum_{n=1}^{m}\left[B_n \sin\left(\frac{n\pi(\lambda-\lambda_i)}{\lambda_f-\lambda_i}\right) + C_n \cos\left(\frac{n\pi(\lambda-\lambda_i)}{\lambda_f-\lambda_i}\right)\right], \quad (1)$$

where $\varepsilon_{Hb}(\lambda)$ and $\varepsilon_{HbO2}(\lambda)$ are the extinction coefficients at wavelength $\lambda$ of deoxyhaemoglobin and oxyhaemoglobin, respectively, $[Hb]$ and $[HbO_2]$ denote the corresponding concentrations, $\lambda_i$ and $\lambda_f$ are, respectively, the shortest and the longest wavelengths in a particular data set, and $A_0$, $B_n$ and $C_n$ are the expansion coefficients. Using the measured values of $\mu_a(\lambda)$ and extinction coefficients for oxyhaemoglobin published by Wray et al (1998), and for deoxyhaemoglobin by Matcher et al (1995), as inputs, the SVD algorithm returns the concentrations of Hb and $HbO_2$ and the amplitudes of the terms that represent the background absorption. The saturation ($SO_2$) is then computed from the concentrations of the haemoglobin species as,

$$SO_2 = [HbO_2]/([HbO_2] + [Hb]).$$

© Cancer Research Campaign 1999

### Statistical treatment of diffuse reflectance data

The uncertainties in the diffuse reflectance data acquired by the CCD camera are calculated using Poisson counting statistics. Uncertainties in the individual values of $\mu_a$ are returned by the non-linear least squares algorithm (Press et al, 1992) that is used to fit the diffusion theory expression described above to the measured diffuse reflectance. In displaying absorption spectra such as those shown in Figure 4(A and B), $\mu_a$s from three adjacent wavelengths are averaged together, and their errors are propagated by conventional means (Bevington, 1969). Errors in the concentrations of oxy- and deoxyhaemoglobin (Figure 5B) are returned by the SVD algorithm (Press et al, 1992). Propagation of these errors is done to determine uncertainties in the $HbO_2$ saturations (Figure 5) and the changes in the $HbO_2$ saturations and total haemoglobin concentrations (Figures 6 and 7).

### Animals and tumours

R3230 mammary adenocarcinomas are serially transplanted subcutaneously in the abdominal region of female Fischer rats (100–120 g). For near infrared spectroscopy experiments, 1 mm × 1 mm × 3 mm tumour sections were implanted using a sterile trochar technique (Hilf et al, 1965). Implantation of this rectangular tissue section produced ellipsoidal tumour volumes that were in general more viable at sizes suitable for spectroscopy than the hemispherical tumours that result from similarly transplanted 1 mm × 1 mm × 1 mm sections. Spectroscopy was performed when tumour dimensions were approximately 20 mm × 12 mm, which, assuming an ellipsoidal geometry, correspond to tumour volumes of approximately 1.5 cc. In order to immobilize the animal and to avoid stress associated with confinement, anaesthetic (75 mg $kg^{-1}$ ketamine hydrochloride and 6 mg $kg^{-1}$ xylazene) was administered prior to data acquisition and was given in smaller doses (0.4 of initial) as needed (approximately every 20 min). Carbogen (5% $CO_2$, 95% $O_2$) or air was administered via a nose cone at a flow rate of 3 l $min^{-1}$. Animal care was conducted according to guidelines established by the University Committee on Animal Resources at the University of Rochester.

### RESULTS

Differences in the absorption spectra of oxy- and deoxyhaemoglobin are the basis for near infrared optical monitoring of haemoglobin oxygen saturation in tissue. Figure 2 presents the spectrum of oxyhaemoglobin (dashed line) reported by Wray et al (1988) and that of deoxyhaemoglobin (solid line) reported by Matcher et al (1995). These spectra were acquired in a conventional absorption spectrophotometer from non-scattering fluid solutions containing haemoglobin extracted from red blood cells. The oxygen content of the solution was established by equilibrating with either 100% $N_2$ or $O_2$ in a tonometer. Other reports of haemoglobin absorption spectra in this wavelength range can be found in the literature; however, we have found that those of Wray et al (for oxyhaemoglobin) and of Matcher et al (for deoxyhaemoglobin) are in close agreement with spectra that we have obtained from intact human erythrocytes using our diffuse reflectance method (Hull et al, 1998). Therefore, we use these published extinction coefficients in analysing our data with Eq (1).

Figure 3 shows representative absorption spectra reconstructed from diffusion theory analysis of diffuse reflectance measurements made on the skin surface directly over an R3230AC tumour in a living, anaesthetized animal. Three cases are presented corresponding to (i) the animal breathing room air, (ii) during carbogen inhalation, and (iii) several minutes after the cessation of carbogen administration. The absorption peak near 760 nm, which is characteristic of deoxyhaemoglobin, was evident in all spectra recorded from tumours during inhalation of room air (solid line in Figure 3). With the onset of carbogen breathing, the amplitude of this peak rapidly decreased, and the qualitative appearance of the spectrum reflected a large oxyhaemoglobin contribution (dashed line). At the end of carbogen delivery, the spectrum gradually returned to its baseline shape; however, the time required for this restoration was quite variable in different animals and was sometimes as long as 16 min.

As noted above, accurate determination of haemoglobin oxygen saturation in tissue requires that the background (non-haemoglobin) absorption is accounted for correctly. We accomplish this by representing the background absorption with an expansion in

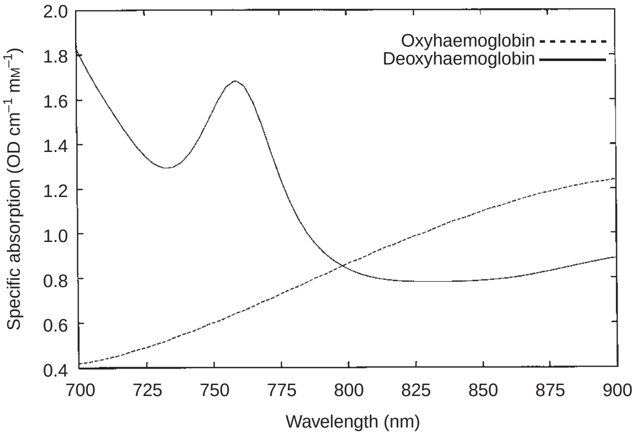

**Figure 2** Near infrared absorption spectra of deoxy- (—) and oxyhaemoglobin (- - -). The oxyhaemoglobin spectrum is from Wray et al (1988); the deoxyhaemoglobin spectrum is from Matcher et al (1995)

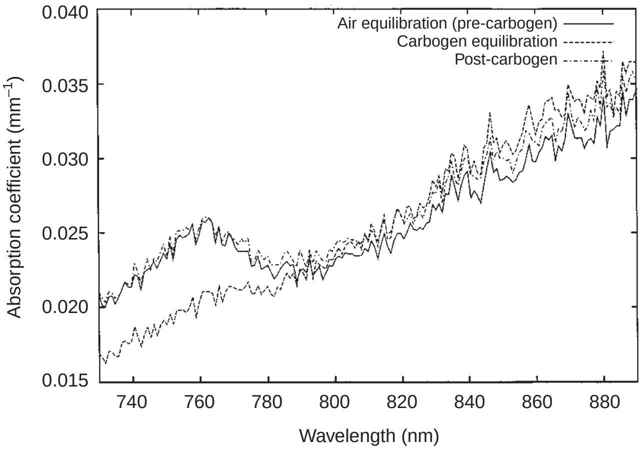

**Figure 3** Absorption spectra reconstructed from diffuse reflectance measurements performed on an R3230AC tumour prior to carbogen delivery (—), during maximum response to carbogen (- - -), and upon restoration of equilibrium after carbogen was stopped (– - –)

© Cancer Research Campaign 1999

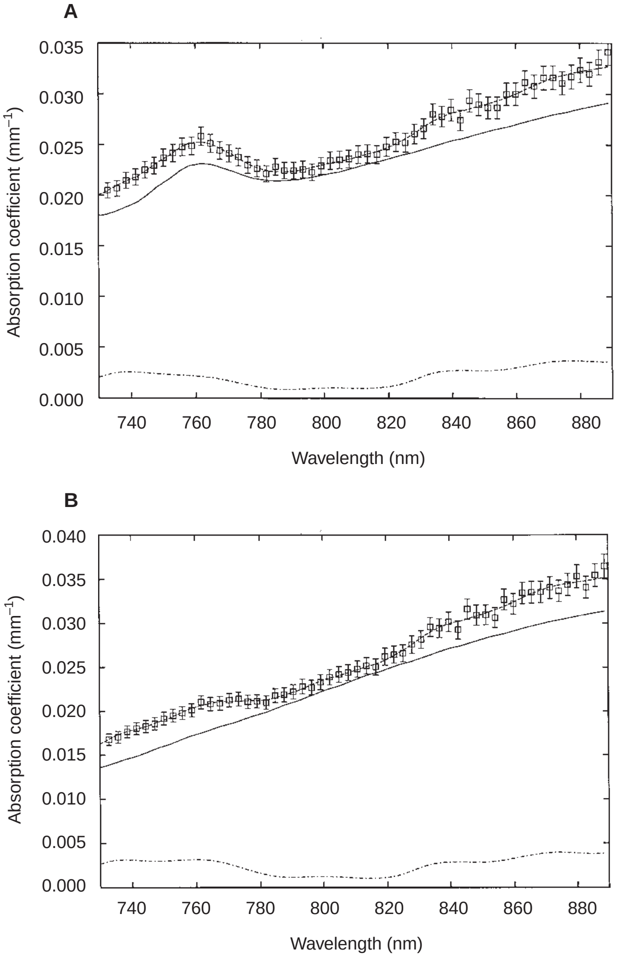

**Figure 4** Results of the singular value decomposition (SVD) analysis (Eq (1)) of two of the spectra shown in Figure 3. The symbols (□) depict the absorption coefficients ($\mu_a$) derived from the diffusion theory analysis of the diffuse reflectance measurements, the solid lines (—) depict the SVD estimate of the contribution resulting from haemoglobin (oxy- plus deoxy-), and the dot-dashed lines (– - –) show the SVD estimates of the tumour absorption that result from chromophores other than haemoglobin. The dashed line (- - -) shows the best fit of Eq (1) to the data. The spectrum in (**A**) was taken prior to carbogen; the spectrum in (**B**) was acquired during maximum response to carbogen. The determination of the error bars is described in Materials and Methods

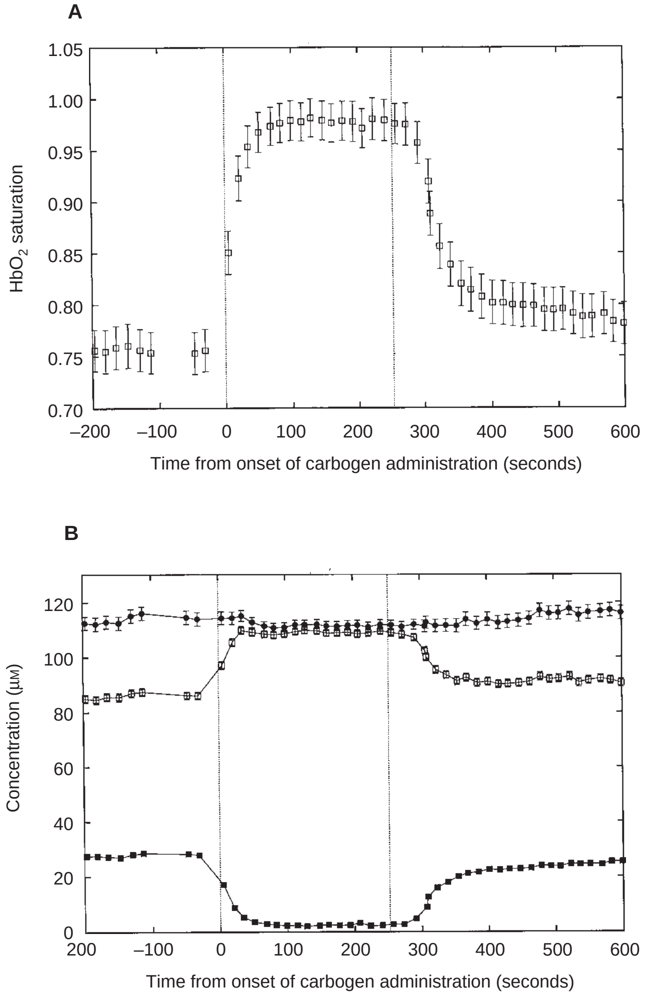

**Figure 5** Haemoglobin oxygen saturation (**A**) and concentrations (**B**) of total haemoglobin (●), oxyhaemoglobin (□), and deoxyhaemoglobin (■) for a single R3230AC tumour before, during and after carbogen breathing. The vertical line at time = 0 corresponds to the beginning of carbogen inhalation; the vertical line at the later time corresponds to the end. The determination of the error bars is described in Materials and Methods sines and cosines, as described in Materials and Methods (Eq (1)). This method requires no prior knowledge of the spectral features of the individual absorbing species, although the SVD algorithm requires that the magnitude of the background represented in this way be small with respect to the haemoglobin absorption. This criterion is met in the spectral range of our experiments in vivo. Figure 4 shows results of applying Eq (1) to the absorption spectra depicted in Figure 3. The spectrum in Figure 4A was obtained while the rat breathed room air, while that in Figure 4B was taken during carbogen inhalation. In both cases, the data points (with uncertainties) are the individual absorption coefficients ($\mu_a$s) obtained from analysis of the diffuse reflectance measurements. The solid lines are the SVD estimate of the absorption from haemoglobin (oxy- plus deoxyhaemoglobin), while the dot-dashed lines are the estimates of the absorbing background. The dashed line through the data points represents the best fit of Eq (1) to the data. Thus, by separating the contribution of haemoglobin to the total absorption coefficient from that of the other chromophores, the method ensures that only the haemoglobin absorption is used in the calculation of the haemoglobin oxygen saturations. We note that, at least in this particular case, the background absorption of the tumour remained approximately unchanged during carbogen administration.

Haemoglobin oxygen saturations determined from data recorded before, during and after carbogen inhalation are shown for a representative tumour in Figure 5A. The vertical, dotted line at time = 0 s indicates the beginning of carbogen breathing; the second vertical line indicates the end. Figure 5B shows separately the concentrations of Hb, $HbO_2$ and total haemoglobin ([Hb] + [$HbO_2$]) for the tumour over the same time course. In this case, the initial $SO_2$ was slightly greater than 0.75. With the onset of carbogen breathing, the saturation rose over a period of approximately 50 s to 0.97, where it remained very constant for the duration of administration. During the period after carbogen was

 © Cancer Research Campaign 1999

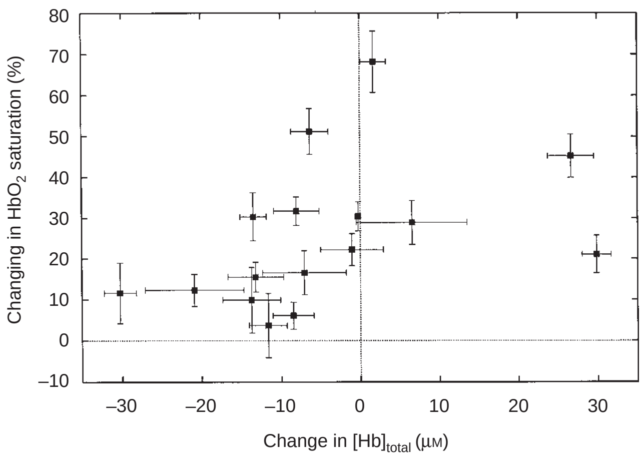

**Figure 6** The carbogen-induced change in haemoglobin oxygen saturation (maximum saturation minus initial saturation) vs the change in the total haemoglobin concentration (haemoglobin concentration at the time of maximum saturation minus initial concentration) for all tumours included in this study ($n$ = 16). The determination of the error bars is described in Materials and Methods

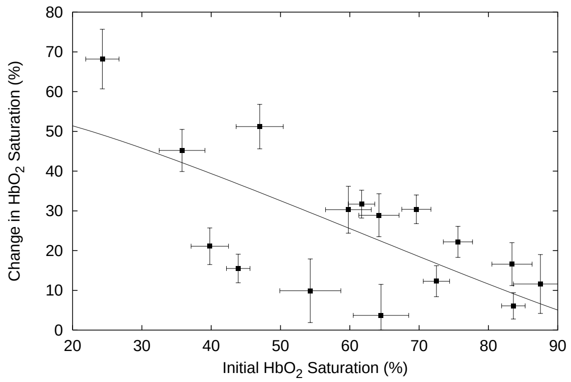

**Figure 7** The maximum carbogen-induced change in haemoglobin oxygen saturation vs the initial saturation for all tumours included in this study ($n$ = 16). The solid line is a fit of Eq (2) to these data. The fit yielded a carbogen-induced change in blood $pO_2$ of 29.7 ± 6.6 torr. The determination of the error bars is described in Materials and Methods stopped, the saturation remained at or near its highest level for 30 s, after which it gradually declined over the next 100 s until reaching an apparent equilibrium, which was slightly elevated with respect to the initial baseline value. This pattern is reflected in the concentrations of Hb (■) and $HbO_2$ (□) plotted in Figure 5B. In this tumour, the total haemoglobin concentration (●) remained approximately constant.

Although a possible mechanism for the observed increase in tumour oxygenation is an increased haemoglobin concentration, which might be expected in response to vasodilation or to the resumption of flow in acutely closed tumour blood vessels, our data demonstrate that such a mechanism was not responsible for the enhanced saturations. In Figure 6, we plot the maximum change in tumour $SO_2$ vs the change in the total haemoglobin concentration for the 16 tumours in the study. The majority (10 of 16) of tumours showed a decreased haemoglobin concentration at the time corresponding to the peak oxygen enhancement. In three tumours the haemoglobin concentration remained approximately constant, while in three others, the concentration increased. Among all tumours, there was no correlation between the magnitude of the change in the haemoglobin concentration (either positive or negative) and the increase in the measured tumour $SO_2$.

The data in Figure 7 compare the maximum change in tumour $SO_2$ with the initial saturation measured immediately prior to carbogen administration. There appear to be roughly two subpopulations of tumours, one of which exhibits a greater degree of change in saturation than the other for a given initial $SO_2$. Within each of these subpopulations, there is a trend toward a larger carbogen-induced change for tumours with lower initial saturation. Using a pH of 7.15 for the R3230AC tumour, which has been determined by Ceckler et al (1991) from $^{31}$P NMR spectroscopy measurements, and the Hill parameters n (2.46) and $p_{50}$ (33.7 torr) corresponding to this pH (Zwart et al, 1982, 1984), we fit an expression of the form

$$\Delta SO_2 = \frac{\left[\left(\frac{SO_{2init}\,p_{50}{}^{n}}{1-SO_{2init}}\right)^{\frac{1}{n}} + \Delta pO_2\right]^{n}}{p_{50}{}^{n} + \left[\left(\frac{SO_{2init}\,p_{50}{}^{n}}{1-SO_{2init}}\right)^{\frac{1}{n}} + \Delta pO_2\right]^{n}} - SO_{2init} \qquad (2)$$

to the data in Figure 7 to find the average change in blood $pO_2$ ($\Delta pO_2$) induced by carbogen. The derivation of this equation is presented in the Appendix. The fit of Eq (2) to the data is shown as the solid line in the Figure, and the carbogen-induced change in $pO_2$ returned by the fit is 29.7 ± 6.6 torr.

### DISCUSSION

Spatially averaged tumour haemoglobin oxygen saturation ($SO_2$) and haemoglobin concentration are reported through non-invasive measurements of near infrared diffuse reflectance. The near infrared measurement samples all vessels within the light field and thus reports an average that is presumably weighted by the relative arterial and venous volumes. In the series of experiments described here, the magnitude of the change in $SO_2$ induced by carbogen was highly variable, although in every tumour ($n$ = 16) we observed some increase in saturation. The rate of increase in the saturation was relatively rapid, with the maximum $SO_2$ typically reached within 1 min after carbogen breathing began. With the termination of carbogen administration, the return to baseline $SO_2$ was slower, sometimes taking as long as 15 min. The mechanism through which carbogen inhalation enhanced the $SO_2$ in this tumour model clearly did not involve increasing the total haemoglobin concentration, as would occur if, for example, the total tumour blood volume increased. Indeed, as is evident from the data shown in Figure 6, in the majority of tumours, the total haemoglobin concentration decreased in response to carbogen breathing. This observation may be a consequence of the 'steal effect', in which vasodilation in the normal circulation results in a loss of blood volume in the tumour, where physiological regulation is deficient or absent. Among the tumours that exhibited decreased haemoglobin volume, the extent of volume change was not related to the magnitude of the increase in the $SO_2$ (Figure 6).

© Cancer Research Campaign 1999

The data suggest a relationship between the extent of the carbogen-induced change in saturation and the initial $SO_2$ of the tumour, with a tendency for the largest induced changes to occur in those tumours with the lowest initial saturations (Figure 7). Qualitatively, one way that this relationship can be understood is on the basis of the shape of the haemoglobin–oxygen dissociation curve (the Hill curve). At the lower oxygen partial pressures where the Hill curve is roughly linear, an increase in partial pressure will give rise to a corresponding increase in the $SO_2$. At higher partial pressures where the slope of the Hill curve becomes more gradual, the same increase in blood oxygen content will produce a lesser effect on the saturation. With respect to the magnitude of this dependence, the data presented in Figure 7 also suggest that there may be two subpopulations of tumours in our study. This finding could be a result of the sensitivity of the Hill curve to pH (Benesch and Bensch, 1974; Zwart et al, 1982), although we have no direct evidence to support this interpretation. In more acidic environments, haemoglobin more readily releases oxygen at a given oxygen partial pressure. Thus, a particular tumour's pH will influence the extent to which an induced change in blood $pO_2$ will be reflected in an increased $SO_2$. Our observations may be explained either by differences in the initial pH in subpopulations of these tumours or by differences in the response to $CO_2$ in these subpopulations.

In addition to the variability in the extent of the carbogen-induced change in tumour $SO_2$, the data presented in Figures 6 and 7 show that the initial (pre-carbogen) $SO_2$ and the carbogen-induced change in total haemoglobin concentration also varied significantly for the 16 tumours in this study. The reasons for this pronounced heterogeneity are not clear. There was no obvious relationship between initial $SO_2$ and tumour volume (data not shown). Although the initial anaesthetic dose administered to the animals was constant, some variation in the time between injection of anaesthetic and the diffuse reflectance measurements was inevitable. The need for smaller subsequent injections to sustain chemical restraint was also variable among animals, and it is possible that one, or the combination of, these anaesthetic-related effects was responsible for some of the observed variability in the data. We note, however, that investigators using other techniques have also reported significant intratumour and intertumour heterogeneity in $SO_2$ within the same tumour type (Fenton et al, 1995 and references therein). Clearly, a detailed appreciation of the physiological information available from near infrared spectroscopy is not yet available, and studies designed to further this understanding appear to be warranted. Comparisons of tumour $SO_2$ determined in vivo using near infrared spectroscopy with similar determinations made using cryospectroscopy (Fenton et al, 1995) would be particularly relevant, in that the latter technique provides haemoglobin saturations in individual vessels in frozen tumour sections.

As we have implemented the technique, near infrared spectroscopy (NIRS) provides a measure of the average haemoglobin $SO_2$ and therefore of the average response to oxygen modifiers such as carbogen in the volume of tissue sampled by the optical probe. It is possible to consider approaches to low resolution imaging of tissue optical properties using near infrared light, which in principle may allow spatially resolved determinations of tumour $SO_2$ (for example, Danen et al, 1998). Whether or not this spectroscopy will be a clinically useful predictor of tumour response to oxygen-dependent interventions such as ionizing radiation therapy and photodynamic therapy remains to be determined. Nevertheless, the method possesses attractive features that make it potentially useful as a research tool and eventually perhaps in some clinical settings. NIRS is completely non-invasive, thereby making it suitable in situations where it is desirable to perform repeated measurements on the same tumour, for example, during a course of fractionated radiation therapy. Its non-invasive character also means that it does not perturb tissue oxygen, either by oxygen consumption or by induction of bleeding, and it is likely to be well-tolerated by patients. The method reports haemoglobin oxygen saturation directly, which will likely be useful in discerning mechanisms of action of various modifiers of tumour oxygenation. Recently, for instance, Robinson et al (1995, 1997) have described changes in gradient-echo magnetic resonance (MR) images in response to carbogen breathing in animal tumour models. The magnetic resonance signal from flowing blood may be influenced by its deoxyhaemoglobin content or by its degree of saturation (i.e. incomplete restoration of equilibrium) from repetitive radiofrequency pulses. Thus, from an enhanced signal in a gradient echo MR image alone it is in general not possible to distinguish between a decreased deoxyhaemoglobin concentration and a reduction in the saturation of the MR signal as a result of increased blood flow velocity (without a change in deoxyhaemoglobin) through the region of interest (Robinson et al, 1997). Because NIRS is capable of determining the $SO_2$ independent of haemodynamic changes, it could be useful in resolving ambiguities like this one. NIRS provides good temporal resolution, with signal acquisition times on our system of approximately 5 s. Finally, the instrumentation required to perform the steady-state form of diffuse reflectance spectroscopy is relatively inexpensive and can be as portable as an ultrasound system, thereby making it possible to consider measurements in the clinic immediately prior to or during therapy.

Of course, the method is not without its own limitations, especially in its current form. Although significantly reduced optical absorption of water and of haemoglobin in the near infrared spectral region (650–900 nm) enables light to penetrate several centimetres in tissue, it is unlikely that NIRS will be useful in the quantitative evaluation of haemoglobin saturation in tumours residing far from a surface that is accessible to an optical fibre probe. Further, the particular form of analysis that we have adopted assumes spatially homogeneous optical properties. This assumption, however, is not fundamental to the approach. Very recent work by other investigators has demonstrated diffusion-theory-based expressions that are capable of extracting optical properties of layered structures, provided that the thickness of the upper layer in contact with the optical probe is not too thick (Kienle et al, 1998). Another limitation imposed by the diffusion theory approximation to light transport is that, in order to recover accurate values of the absorption coefficients, relatively large (approximately 15 mm) separations are required between the optical fibre that delivers light to the tissue and the most remote detection fibre. Although this requirement precludes the use of this technique on smaller tumours at present, it is not fundamental to the optical method and may be relaxed using other theoretical approaches. Investigation of suitable alternatives is an active area of research in several laboratories.

The need for improved methods of monitoring tumour oxygenation in patients continues to be recognized (Saunders and Dische, 1996; Höckel et al, 1993*b*). Even with its current limitations, the

*© Cancer Research Campaign 1999*

ability of NIRS to determine $SO_2$ non-invasively, and to monitor changes induced by carbogen inhalation, is sufficiently encouraging that its use could be considered in several anatomic sites where hypoxia has been recognized as a significant factor influencing the resistance of tumours to ionizing radiation therapy. These sites include the cervix, head and neck, superficial regions of the breast, and superficial soft tissue in the extremities.

### ACKNOWLEDGEMENTS

This research was supported by USPHS grants CA68409 and CA36856. We gratefully acknowledge the Animal Tumor Research Facility of the University of Rochester Cancer Center (CA11198) for maintaining and transplanting the R3230AC tumour line, and we thank Mr Jim Havens for assistance in tumouring and animal handling.

### REFERENCES

Benesch RE and Bensch R (1974) The mechanism of interaction of red cell organic phosphates with hemoglobin. *Adv Protein Chem* **28**: 211–237

Bevington PR (1969) *Data Reduction and Error Analysis for the Physical Sciences.* McGraw-Hill: New York

Brizel DM, Lin S, Johnson JL, Brooks J, Dewhirst MW and Piantadosi CA (1995) The mechanisms by which hyperbaric oxygen and carbogen improve tumour oxygenation. *Br J Cancer* **75**: 1120–1124

Brizel DM, Scully SP, Harrelson JM, Layfield LJ, Bean JM, Prosnitz LR and Dewhirst MW (1996) Tumor oxygenation predicts for the likelihood of distant metastases in human soft tissue sarcoma. *Cancer Res* **56**: 941–943

Brizel DM, Sibley GS, Prosnitz LR, Scher RL and Dewhirst MW (1997) Tumor hypoxia adversely affects the prognosis of carcinoma of the head and neck. *Int J Radiat Oncol Biol Phys* **38**: 285–289

Ceckler TL, Gibson SL, Kennedy SD, Hilf R and Bryant RG (1991) Heterogeneous tumour response to photodynamic therapy assessed by in vivo localised $^{31}P$ NMR spectroscopy. *Br J Cancer* **63**: 916–922

Danen RM, Wang Y, Li XD, Thayer WS and Yodh AG (1998) Regional imager for low-resolution functional imaging of the brain with diffusing near-infrared light. *Photochem Photobiol* **67**: 33–40

Fenton BM, Raubertas RF and Boyce DJ (1995) Quantification of micro-regional heterogeneities in tumour oxygenation using intravascular $HbO_2$ saturations. *Radiat Res* **141**: 49–56

Graeber TG, Osmanian C, Jacks T, Housman DE, Koch CJ, Lowe SW and Giacca AJ (1996) Hypoxia-mediated selection of cells with diminished apoptotic potential in solid tumours. *Nature* **379**: 88–91

Grau C, Horsman MR and Overgaard J (1992) Improving the radiation response in a C3H mouse mammary carcinoma by normobaric oxygen or carbogen breathing. *Int J Radiat Oncol Biol Phys* **22**: 415–419

Haskell RC, Svaasand LO, Tsay T, Feng T, McAdams MS and Tromberg BJ (1994) Boundary conditions for the diffusion equation in radiative transfer. *J Opt Soc Am A* **11**: 2727–2741

Hilf R, Michel I, Bell C, Freeman JJ and Borman A (1965) Biochemical and morphological properties of a new lactating tumour line in the rat. *Cancer Res* **25**: 286–299

Hill RP and Bush RS (1977) Dose fractionation studies with a murine sarcoma under conditions of air or carbogen (95% $O_2$ + 5% $CO_2$) breathing. *Int J Radiat Oncol Biol Phys* **2**: 913–919

Höckel M, Knoop C, Schlenger K, Vorndran B, Bauβmann E, Mitze M, Knapstein PG and Vaupel P (1993*a*) Intratumoral $pO_2$ predicts survival in advanced cancer of the uterine cervix. *Radiother Oncol* **26**: 45–50

Höckel, M, Vorndran, B, Schlenger K, Baußmann E and Knapstein PG (1993b) Tumor oxygenation: a new predictive parameter in locally advanced cancer of the uterine cervix. *Gynecol Oncol* **51**: 141–149

Horsman MR, Chaplin DJ and Brown JM (1989) Tumor radiosensitization by nicotinamide: a result of improved perfusion and oxygenation. *Radiat Res* **118**: 139–150

Hull EL and Foster TH (1997) Noninvasive near-infrared hemoglobin spectroscopy for in vivo monitoring of tumor oxygenation and response to oxygen modifiers. In *Optical Tomography and Spectroscopy of Tissue*: *Theory, Instrumentation, Model, and Human Studies II*, Chance B and Alfano RR (eds), pp. 355–364. *SPIE Proc* **2979**: Bellingham

Hull EL, Nichols MG and Foster TH (1998) Quantitative broadband near-infrared spectroscopy of tissue-simulating phantoms containing erythrocytes. *Phys Med Biol* **43**: 3381–3404

Kienle A and Patterson MS (1997) Improved solutions of the steady-state and the time-resolved diffusion equations for reflectance from a semi-infinite turbid medium. *J Opt Soc Am A* **14**: 246–254

Kienle A, Patterson MS, Dögnitz N, Bays R, Wagnières G and van den Bergh H (1998) Non-invasive determination of the optical properties of two-layered turbid media. *Appl Opt* **37**: 779–791.

Matcher SJ, Elwell CE, Cooper CE, Cope M and Delpy DT (1995) Performance comparison of several published tissue near-infrared spectroscopy algorithms. *Anal Biochem* **227**: 54–68

Nichols MG, Hull EL and Foster TH (1997) Design and testing of a white light steady-state diffuse reflectance spectrometer for determination of optical properties of highly scattering systems. *Appl Opt* **36**: 93–104

Okunieff P, Hoeckel M, Dunphy EP, Schlenger K, Knoop C and Vaupel P (1993) Oxygen tension distributions are sufficient to explain the local response of human breast tumors treated with radiation alone. *Int J Radiat Oncol Biol Phys* **26**: 631–636

Olive PL and Inch WR (1973) Effect of inhaling oxygen/carbon dioxide mixtures on oxygenation and cure by X-rays of the C3HBA mouse mammary carcinoma. *Radiology* **106**: 673–678

Press WH, Teukolsky SA, Vetterling WT and Flannery BP (1992) *Numerical Recipes in C*: *The Art of Scientific Computing*. Cambridge University Press: New York

Robinson SP, Howe FA and Griffiths JR (1995) Non-invasive monitoring of carbogen-induced changes in tumor blood flow and oxygenation by functional magnetic resonance imaging. *Int J Radiat Oncol Biol Phys* **33**: 855–859

Robinson SP, Rodrigues LM, Ojugo ASE, McSheehy PMJ, Howe FA and Griffiths JR (1997) The response to carbogen breathing in experimental tumor models monitored by gradient-recalled echo magnetic resonance imaging. *Br J Cancer* **75**: 1000–1006

Rojas A (1992) ARCON: accelerated radiotherapy with carbogen and nicotinamide. *Br J Radiol* **24**: 174–178

Saunders M and Dische S (1996) Clinical results of hypoxic cell radiosensitization from hyperbaric oxygen to accelerated radiotherapy, carbogen and nicotinamide. *Br J Cancer* **74**: (Suppl. XXVII): S271–S278

Stone HB, Brown JM, Phillips TL and Sutherland RM (1993) Oxygen in human tumors: correlations between methods of measurement and response to therapy. *Radiat Res* **136**: 422–434

Thomlinson RH (1960) An experimental method for comparing treatments of intact malignant tumours in animals and its application to the use of oxygen in radiotherapy. *Br J Cancer* **14**: 555–576

Vaupel P, Schlenger K, Knoop C and Höckel M (1991) Oxygenation of human tumors: evaluation of tissue oxygen distribution in breast cancers by computerized $O_2$ tension measurements. *Cancer Res* **51**: 3316–3322

Voûte PA, van der Kleij AJ, De Kraker J, Hoefnagel CA, Tiel-van Buul MMC and Van Gennip H (1995) Clinical experience with radiation enhancement by hyperbaric oxygen in children with recurrent neuroblastoma stage IV. *Eur J Cancer* **31A**: 596–600

Wilson BC, Farrell TJ and Patterson MS (1990) An optical fiber-based diffuse reflectance spectrometer for non-invasive investigation of photodynamic sensitizers in vivo. In *Future Directions and Applications in Photodynamic Therapy*, Gomer CJ (ed), pp. 219–232. *SPIE Proc*. **IS6**: Bellingham

Wray S, Cope M, Delpy DT, Wyatt JS and Reynolds EOR (1988) Characterization of the near infrared absorption spectra of cytochrome $aa_3$ and haemoglobin for the non-invasive monitoring of cerebral oxygenation. *Biochim Biophys Acta* **933**: 184–192

Zwart A, Kwant, G, Oeseburg B and Zijlstra WG (1982) Oxygen dissociation curves for whole blood, recorded with an instrument that continuously measures $p_{O2}$ and $S_{O2}$ independently at constant t, $P_{CO2}$, and pH. *Clin Chem* **28**: 1287–1292.

Zwart A, Kwant, G, Oeseburg B and Zijlstra WG (1984) Human whole-blood oxygen affinity: effect of temperature. *J Appl Physiol*: *Respir Environ Exercise Physiol* **57**: 429–434

### APPENDIX

The Hill equation describing the haemoglobin–oxygen dissociation curve is written,

© Cancer Research Campaign 1999

$$SO_2 = \frac{pO_2^n}{p_{50}^n + pO_2^n} \quad \text{(A1)}$$

where $SO_2$ is the haemoglobin oxygen saturation, $pO_2$ is the oxygen partial pressure, $p_{50}$ is the oxygen partial pressure at which the $SO_2$ is 0.5, and n is the Hill coefficient. The pH of the R3230AC tumour is approximately 7.15, as determined by Ceckler et al (1991) using $^{31}$P NMR spectroscopy. The Hill coefficient, n and the $p_{50}$ corresponding to this pH are 2.46 and 33.7 torr, respectively (Zwart et al, 1982, 1984). In our experiments we measure the initial $SO_2$ (prior to carbogen delivery), which we denote $SO_{2init}$, and the final $SO_2$ after full equilibration on carbogen, which we denote here as $SO_{2f}$. The change in saturations, $\Delta SO_2$, is written simply

$$\Delta SO_2 = SO_{2f} - SO_{2init}. \quad \text{(A2)}$$

Writing $SO_{2f}$ in terms of the corresponding $pO_{2f}$ through Eq (A1), this becomes

$$\Delta SO_2 = \frac{pO_{2f}^n}{p_{50}^n + pO_{2f}^n} - SO_{2init}. \quad \text{(A3)}$$

The $pO_{2f}$ is the sum of the initial $pO_2$ and the carbogen-induced change, $\Delta pO_2$. Expressing the initial $pO_2$ in terms of the initial $SO_2$ through Eq (A1), we can write $pO_{2f}$ as,

$$pO_{2f} = \left(\frac{SO_{2init}\, p_{50}^n}{1 - SO_{2init}}\right)^{\frac{1}{n}} + \Delta pO_2. \quad \text{(A4)}$$

Substitution of the right-hand side of (A4) into Eq (A3) yields

$$\Delta SO_2 = \frac{\left[\left(\frac{SO_{2init}\, p_{50}{}^n}{1 - SO_{2init}}\right)^{\frac{1}{n}} + \Delta pO_2\right]^n}{p_{50}{}^n + \left[\left(\frac{SO_{2init}\, p_{50}{}^n}{1 - SO_{2init}}\right)^{\frac{1}{n}} + \Delta pO_2\right]^n} - SO_{2init}, \quad \text{(A5)}$$

which is the function that is fit to the data in Figure 7 to extract the carbogen-induced change in blood $pO_2$, $\Delta pO_2$.

*© Cancer Research Campaign 1999*
